# In-Depth Temporal Transcriptome Profiling of an Alphaherpesvirus Using Nanopore Sequencing

**DOI:** 10.3390/v14061289

**Published:** 2022-06-13

**Authors:** Dóra Tombácz, Balázs Kakuk, Gábor Torma, Zsolt Csabai, Gábor Gulyás, Vivien Tamás, Zoltán Zádori, Victoria A. Jefferson, Florencia Meyer, Zsolt Boldogkői

**Affiliations:** 1 Department of Medical Biology, Albert Szent-Györgyi Medical School, University of Szeged, Somogyi u. 4, 6720 Szeged, Hungary; tombacz.dora@med.u-szeged.hu (D.T.); kakuk.balazs@med.u-szeged.hu (B.K.); torma.gabor@med.u-szeged.hu (G.T.); csabai.zsolt@med.u-szeged.hu (Z.C.); gulyas.gabor@med.u-szeged.hu (G.G.); 2 Institute for Veterinary Medical Research, Centre for Agricultural Research, Hungária krt. 21, 1143 Budapest, Hungary; tamas.vivien@agrar.mta.hu (V.T.); zadori.zoltan@agrar.mta.hu (Z.Z.); 3 Department of Biochemistry & Molecular Biology, Entomology & Plant Pathology, Mississippi State University, 408 Dorman P.O. Box 9655, 32 Creelman St., Starkville, MS 39762, USA; vaj13@msstate.edu (V.A.J.); florencia.meyer@msstate.edu (F.M.)

**Keywords:** herpesviruses, bovine alphaherpesvirus type 1, transcriptome, transcript isoforms, long-read sequencing, nanopore sequencing, direct cDNA sequencing, transcription start site, transcription end site

## Abstract

In this work, a long-read sequencing (LRS) technique based on the Oxford Nanopore Technology MinION platform was used for quantifying and kinetic characterization of the poly(A) fraction of bovine alphaherpesvirus type 1 (BoHV-1) lytic transcriptome across a 12-h infection period. Amplification-based LRS techniques frequently generate artefactual transcription reads and are biased towards the production of shorter amplicons. To avoid these undesired effects, we applied direct cDNA sequencing, an amplification-free technique. Here, we show that a single promoter can produce multiple transcription start sites whose distribution patterns differ among the viral genes but are similar in the same gene at different timepoints. Our investigations revealed that the *circ* gene is expressed with immediate–early (IE) kinetics by utilizing a special mechanism based on the use of the promoter of another IE gene (*bicp4*) for the transcriptional control. Furthermore, we detected an overlap between the initiation of DNA replication and the transcription from the *bicp22* gene, which suggests an interaction between the two molecular machineries. This study developed a generally applicable LRS-based method for the time-course characterization of transcriptomes of any organism.

## 1. Introduction

Bovine alphaherpesvirus 1.1 (BoHV-1) is an enveloped virus with a large (~136 kbp) double-stranded DNA genome. BoHV-1 is one of several pathogens of cattle and related ruminants that contribute to bovine respiratory disease, which leads to significant economic losses in the cattle industry worldwide [1]. The DNA sequence of BoHV-1 genome was determined in 1995, using sequences from several viral strains and subtypes [2,3]. More recently, genomes of individual strains have also been sequenced [4,5].

Our previous study has provided the detailed transcriptomic atlas of BoHV-1 [6]. Viral gene expression is sequentially coordinated throughout the lytic infection. Similar to other herpesviruses, BoHV-1 genes can also be classified as immediate–early (IE), early (E), early–late (L1) and late (L2), depending on the expression kinetics throughout the viral replication cycle [7]. The following three BoHV-1 IE genes have been identified: *bicp4*, *bicp0*, and *bicp22*, which are homologues of the herpes simplex virus type 1 (HSV-1) *icp4*, *icp0*, and *icp22* genes, respectively [8]. IE gene expression is activated by a virion component, VP16 (α-TIF), in cooperation with cellular transcription factors [9]. IE proteins bICP4 and bICP0 control the transcription of E genes, which encode enzymes needed for the synthesis of viral DNA [10]. The *us1* gene has also been described as a transcription regulator [11,12]. Promoters of the L gene are also activated by these viral transcription factors [8,13]. The L genes encode the structural elements of the virus, and their expression culminates in virion assembly and egress. Herpesvirus genes tend to be organized into tandem gene clusters, which encode parallel overlapping transcripts sharing the 3′-termini in the following order: ‘abcd’, ‘bcd’, ‘cd’, and ‘d’, where ‘a’ is the most upstream and ‘d’ is the most downstream gene. According to our current knowledge, the downstream coding sequences (CDS) on these multigenic transcripts, with few exceptions [14], are untranslated. Similar to other alphaherpesviruses, BoHV-1 can also enter latency in the peripheral nervous system of the host [15].

Quantitative RT-PCR and microarray approaches are able to detect only the aggregate transcriptional activity of particular genomic regions, but they are unable to distinguish between the parallel-overlapping RNA molecules, splice isoforms, transcript length variants, and multigenic transcripts. Short-read sequencing (SRS) technology has transformed the landscape of the transcriptome research as it enables the analysis of a large number of nucleic acid fragments simultaneously at a relatively low cost. However, this method has some drawbacks, which are mainly related to the length of the reads [16]. SRS alone is known to be inaccurate for the determination of alternative transcription start sites (TSSs), as assembly of sequencing reads extends transcript contigs to the most upstream TSS and therefore lacks the information on internal transcription initiation [17]. For the accurate transcriptome-wide TSS annotations, SRS is combined with other methods, such as CAGE-seq, PRO-seq, and RAMPAGE [18,19,20]. In addition, methods that use PCR generally produce false transcription reads and are biased to the generation of short amplicons, whereas the reads generated by native RNA sequencing lack 15–30 base pairs (bps) from their 5′-ends due to the release of the RNA by the ratcheting molecule before passing through the pore [6]. These undesired shortcomings can be avoided by using an amplification-free direct cDNA sequencing technique.

In the past couple of years, long-read sequencing (LRS) technologies have provided an alternative approach that eludes the limitations of SRS methods. The major advantages of LRS techniques over the SRS approach are that they are able to generate full-length transcripts without the need of assembly algorithms and efficiently identify transcript isoforms and transcriptional overlaps. Additionally, the applied reverse transcription (RT) method applies a template switching mechanism during cDNA synthesis, therefore the LRS approaches can be used for the annotation of TSSs and the transcription end sites (TESs) of the RNA molecules in a single run without the need of additional techniques. Besides Pacific Biosciences (PacBio) and Oxford Nanopore Technologies (ONT) platforms, Loop Genomics has recently developed an LRS approach based on single molecule synthetic long-read sequencing (LoopSeq) [6]. In recent times, single cell [21] and single nuclei methods [22] have been developed for more refined transcriptomic characterizations.

Lately, LRS techniques have been widely applied for the transcriptome analysis of a variety of organisms [23,24,25,26,27,28,29,30,31,32,33,34,35], including herpesviruses [36,37,38,39,40,41,42,43]. These approaches have revealed a far more complex transcriptional landscape than previously anticipated [33]. For example, in many genes, the canonical promoters are accompanied by alternative ones located at a certain distance from the core promoter sequences. The usage of different TSSs is a well-known, cell-type-specific mechanism for generating protein diversity in eukaryotic organisms [44]. The widespread occurrence of alternative TSSs in various viruses has been described in several studies using mainly LRS approaches [45,46,47,48]. A significant proportion of alternative TSSs has been detected within the coding region of the viral genes, which raises the possibilities that these 5′-deleted transcripts might encode N-terminally truncated proteins. It has been previously assumed that the TATA box of promoters initiates the transcription from a single nucleotide [49]. However, recent studies have demonstrated a high complexity of transcription initiation events within the RNA Polymerase II core promoters in mammalian cells [50,51,52]. Upstream open reading frames (uORFs) located at the 5′-untranslated region (5′-UTR) of many transcripts extend the coding capacity of the genes by various mechanisms [40].

The kinetic characterization of herpesvirus gene expression has already been carried out using PacBio platform [53].

In a previous study, we reported the assembly of the BoHV-1 transcriptome atlas using a dual nanopore/synthetic LRS approach [6]. BoHV-1 transcripts were identified by the detection of TSSs, TESs, and splice sites using the LoRTIA software suite developed in our laboratory [40,41]. Only those LoRTIA transcripts that were detected by at least three independent sequencing techniques were accepted as true transcripts. The RT and the synthesis of the second DNA strand often lead to template switching [54]. Furthermore, the oligo(dT) primers can occasionally bind to the A-rich regions of the RNA or to the first cDNA strand, thereby producing false TESs and truncated 3′-ends [55]. Such products were eliminated from further analysis using the new version of the LoRTIA software. In our previous work we have found that the entire viral genome is transcriptionally active. However, we could not identify full-length LoRTIA transcripts from a 336-bp region between the *circ* and *ul54* genes, and from a 1638-bp genomic segment between the *bICP4* and *ORIS-RNA1* genes. In our current study, we carried out a time-lapse analysis of BoHV-1 transcription using direct cDNA sequencing (dcDNA-Seq) on the ONT MinION platform. Applying this method, we were able to monitor the time course utilization of the TSSs and TESs, the expression of BoHV-1 transcripts, and to carry out their kinetic categorization.

## 2. Materials and Methods

### 2.1. Cells and Viruses

Madin–Darby Bovine Kidney (MDBK) cells were infected with the Cooper isolate (GenBank Accession # JX898220.1) of Bovine Herpesvirus 1.1. Each time-point and mock infection consisted of three biological replicates. Cells were incubated at 37 °C in a humidified incubator with 5% CO2 and were cultured with Dulbecco’s modified Eagle’s medium (DMEM) supplemented with 5% (*v*/*v*) fetal bovine serum, 100 U/mL penicillin, and 100 µg/mL streptomycin. The virus stock solutions were prepared by infecting cells with 0.1 multiplicity of infection [MOI = multiplicity of infection, which equates to plaque-forming units (pfu)/cell]. Viral infection was allowed to progress until complete cytopathic effect was observed. The supernatant was collected, and the cellular fraction was subjected to three successive cycles of freezing and thawing in order to release additional intracellular virions. For the kinetic analysis, we used 5 MOI to minimize the number of cells that remain uninfected to avoid the re-initialization of viral cycle in uninfected cells, which would make our analysis uninterpretable. In addition, cells were first incubated at 4 °C for one hour for synchronization of infection, and then placed in a 5% CO2 incubator at 37 °C. Infected cells were collected at 1, 2, 4, 6, 8, and 12 h post infection (p.i.). Cells were washed with phosphate-buffered saline (PBS), scraped from the culture plate, and centrifuged at 3000 RPM for 5 min at 4 °C.

### 2.2. Cycloheximide Treatment

MDBK cells were propagated in DMEM containing 10% fetal bovine serum until 60–70% confluency was reached. Next, the culture medium was replaced by 5 mL serum-free DMEM supplemented with 20 or 100 ug/mL cycloheximide (CHX). After 1 h incubation, the culture medium was replaced by 2 mL 10 MOI virus solution containing the same concentrations of CHX and incubated for 6 h or 8 h. CHX-treated cultures were washed once with PBS, scraped from the dish, and centrifuged at 2000 g for 2 min. The supernatant was removed and the cells were put on dry ice until further use.

### 2.3. RNA Purification

RNA from the viral infected and uninfected cells (six time points, three biological replicates), as well as from the CHX-treated samples, were purified using the spin column-based NucleoSpin RNA kit (Machery-Nagel, Bethlehem, PA, USA), as described in the manual. The following modifications were carried out: (1) proteinase K (0.37 mg/mL final concentration) was added to the samples at the lysis step; (2) TURBO DNA-free™ Kit (Invitrogen) was used to eliminate the potential residual genomic DNA from the isolated RNA samples. The RNA concentration was determined by using Qubit 4.0 Fluorometer and Qubit RNA BR (Broad-Range) Assay Kit (Invitrogen). The quality of RNA was assessed based on RIN values using TapeStation 4150 system (Agilent). RIN scores ≥ 9.3 were used for sequencing.

### 2.4. Poly(A) RNA *Isolation*

The polyadenylated fractions from the RNA samples were extracted using Oligotex mRNA Mini Kit (Qiagen). The kit’s manual was followed; briefly, the volume of the RNA samples was set to 250 µL RNase-free water and it was mixed with 250 µL OBB buffer and 15 µL Oligotex suspension (both from the Qiagen kit). These mRNA–Oligotex mixtures were incubated at 70 °C for 3 min and then at 25 °C for 10 min. The samples were centrifuged at 14,000× g for 2 min, and then the supernatants were discarded. The samples were resuspended in 400 µL OW2 wash buffer (from the kit) and loaded onto the Qiagen spin columns and centrifuged for 1 min at 14,000× g. This washing step was repeated once, and finally, the poly(A)+ RNAs were eluted from the membrane by adding 50 µL hot elution buffer (Qiagen kit). The concentration of the purified poly(A)+ RNA samples were checked by Qubit 4.0 and Qubit RNA HS (High Sensitivity) Assay Kit (Invitrogen).

### 2.5. Direct cDNA *Sequencing*

ONT’s direct (d)cDNA Sequencing Kit (SQK-DCS109) and the dcDNA protocol (ONT) was used to generate libraries from the poly(A)+ RNA samples (100 ng from each) according to the manufacturer’s recommendations. First, a reverse transcription step was carried out using Maxima H Minus Reverse Transcriptase enzyme (Thermo Fisher Scientific) and SSP and VN primers (supplied in the ONT kit). This step was followed by the removal of the potential RNA using RNase Cocktail Enzyme Mix (Thermo Fisher Scientific). For the synthesis of the second cDNA strand, LongAmp Taq Master Mix (New England Biolabs) was used. The end-repair was carried out using NEBNext Ultra II End repair/dA-tailing Module (New England Biolabs) and was followed by the adapter (AMX) ligation using NEB Blunt/TA Ligase Master Mix (New England Biolabs). Each library was barcoded using Native Barcoding Kit (ONT) as described in the manual (Table 1). Mock-infected samples and libraries from the earlier time points were run separately from the later time points in order to avoid the potential “barcode hopping”. Agencourt AMPure XP magnetic beads (Beckman Coulter) were used for purification of the samples following each enzymatic step of the protocol. The concentrations of the cDNAs and dcDNA libraries were measured using Qubit 4.0 and the Qubit dsDNA HS Assay Kit (Invitrogen).

### 2.6. Pre-Processing and Data Analysis

**Figure 1:**
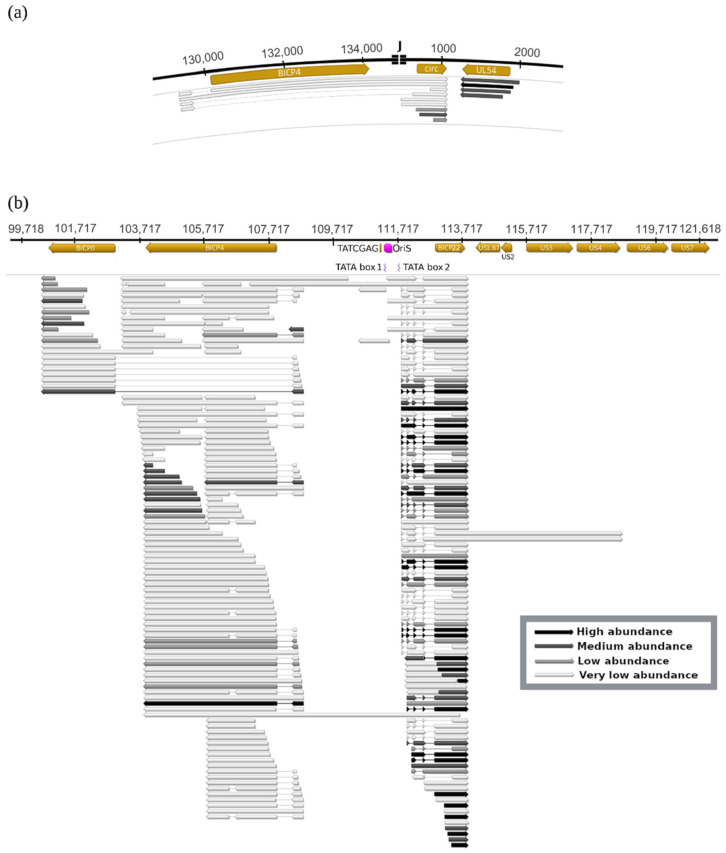
BoHV-1 transcripts generated from the genomic locations of IE genes. (**a**) In the circular BoHV-1 genome, the bicp4 gene occasionally produces both spliced and unspliced readthrough transcripts, which, besides the *bicp4* gene, contain the *circ* gene located at the other terminus of the linear viral genome. Thus, the *circ* gene expression is partly under the control of the immediate–early *bicp4* promoter. (**b**) The three immediate–early genes produce a large variety of spliced and unspliced transcripts. The *bicp4* gene codes for 3′-truncated non-coding RNAs and alternative TESs as well. The expression of a significant fraction of *bicp0* transcripts is controlled by the *icp4* promoter. The *bicp22* gene exhibits the most complex expression pattern among the BoHV-1 genes, due to its use of multiple splice sites, TSSs and TESs, as well as very long transcriptional read-throughs. TATA box2 is the basal promoter of the canonical transcripts, while TATA box1 is located within the OriS. The TAATCGAT sequence is supposed to be the VP16 binding site of the promoter. Transcript abundances are indicated by color code in the figure: as the two extremes, black arrows indicate high abundance, whereas white arrows indicate low abundance transcripts.

**Figure 2:**
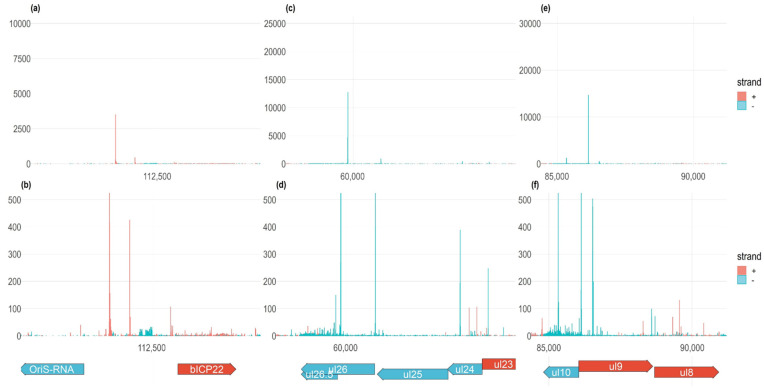
Examples for the alternative transcription start sites. (**a**,**b**) The *bicp22* gene encodes a highly complex TSS pattern. There is an abundant canonical and a somewhat less abundant alternative TSS, and several rare TSSs. (**c**,**d**) Transcripts of *ul26.5-26* genes are illustrated by using the original abundance without setting a limit, (B) and an abundance restricted to 500 TSSs (C). The *ul26.5* gene is transcribed at a higher level than the *ul26* gene. (**e**,**f**) Transcripts of *ul10* gene are illustrated by using the original abundance (D) and an abundance restricted to 500 TSSs (E). Besides the canonical TSS, *ul10* is also expressed to contain two alternative TSSs. All examples are from the 12 hpi samples.

**Figure 3:**
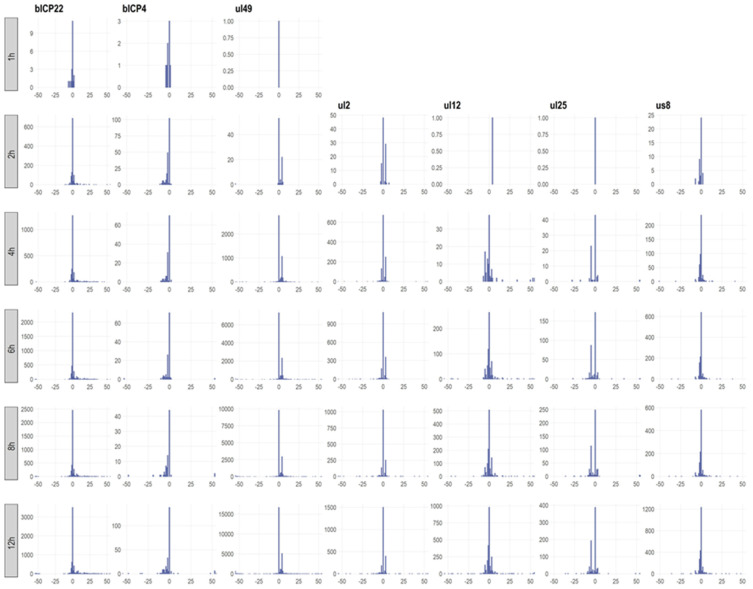
Dynamics of the transcription start site clusters. Promoters initiate transcription from multiple points with a dominant (canonical) TSS. Different promoters produce distinct patterns of TSS clusters, but a promoter generates similar TSS cluster compositions throughout the replication cycle of the virus.

**Figure 4:**
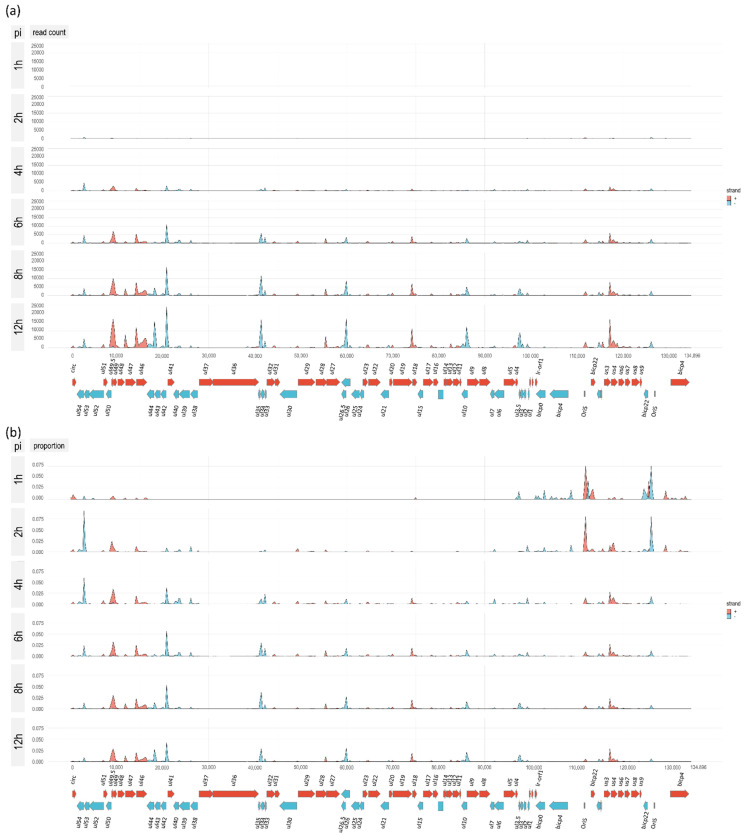
Time-course genome-wide expression of TSSs. This figure shows the TSS distribution along the BoHV-1 genome in a time-varying manner using a density plot. (**a**) The *Y*-axis shows the number of TSSs using the same scales. Red color indicates left-to-right orientation of the TSSs, while blue color indicates the opposite orientation of the TSSs and of course, the same is true for the corresponding genes. (**b**) This panel shows the proportion of TSSs within the total TSS. While the number of TSSs can be reliably compared for the same TSS at different time points, the different TSSs cannot be compared due to the size preference of sequencing.

**Figure 5:**
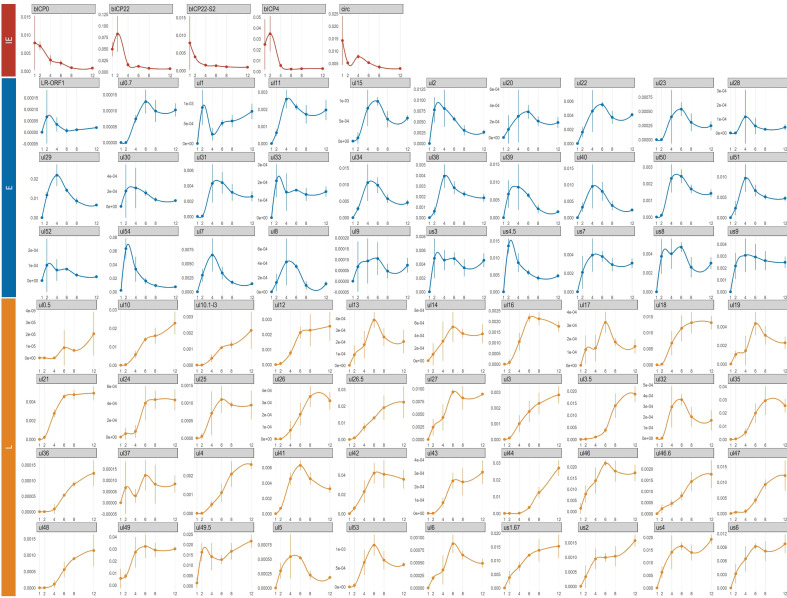
Expression dynamics of canonical and high-abundance non-canonical TSSs. This figure shows the expression dynamics of TSSs throughout the course of viral infection. TSSs and the corresponding transcripts can be categorized on the basis of the curve shape. Red color indicates IE, blue color indicates E, while brown color indicates L transcription kinetics.

**Figure 6:**
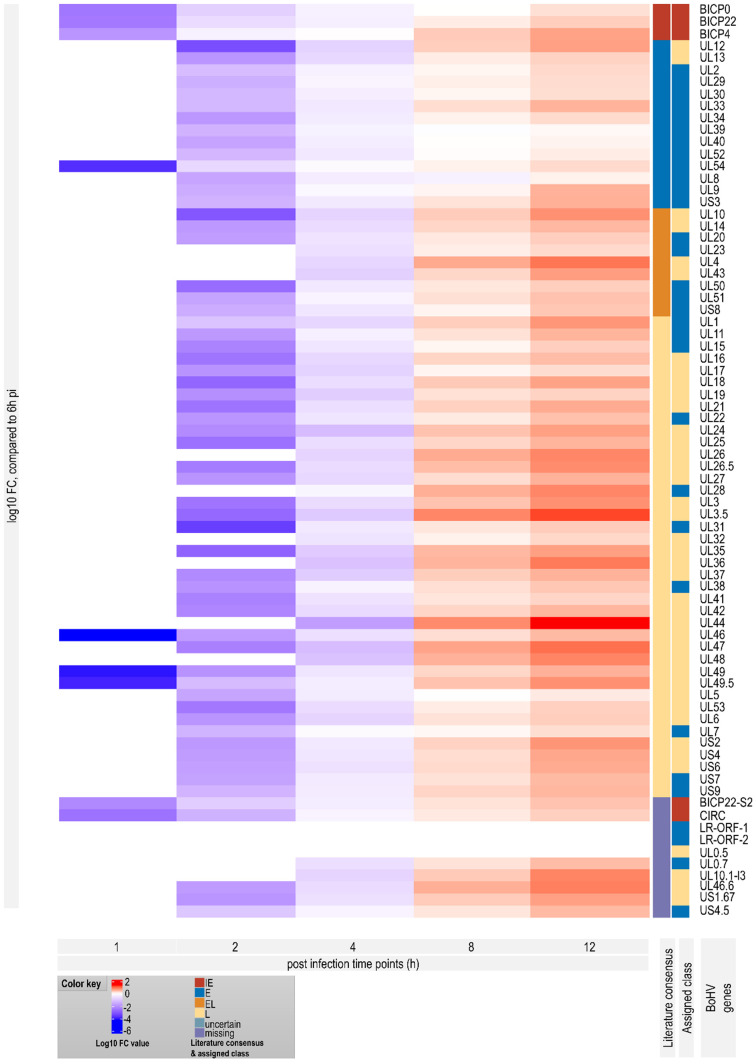
Heatmap of normalized gene expression of Bovine Herpesvirus during infection. Heatmap of Fold Change (log10FC), compared to 6 hpi of the viral genes, according to the abundance of their TSSs. The colors show the mean log10 fold change values of the simple ratio normalized viral read counts compared to 6 hpi samples. For this reason, the 6 hpi sample is omitted. The two annotation columns on the right show the kinetic categories of each gene: the inner column shows the literature consensus (the kinetic category associated most of the time with the gene), while the outer column shows kinetic category according to our results.

**Figure 7:**
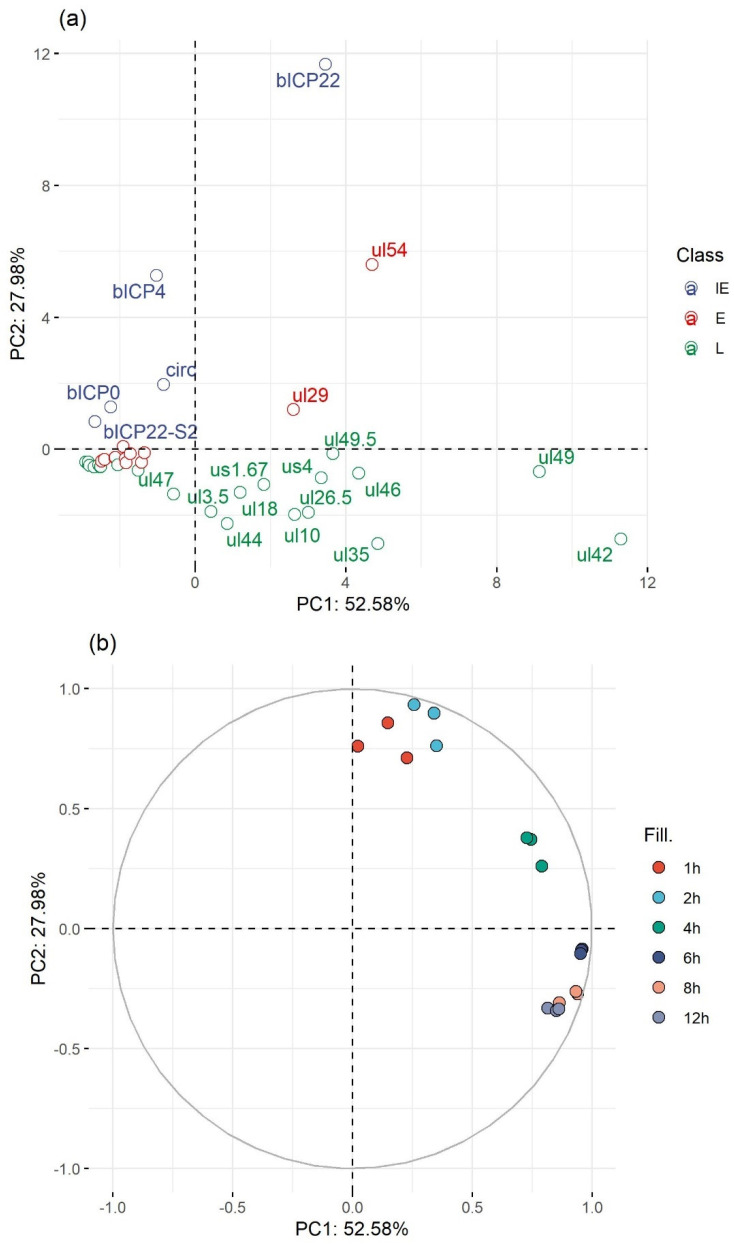
Principal component analysis. PCA of gene abundances (based on TSS counts) normalized to the total viral read count. This exploratory multivariate data analysis method decreases the number of dimensions in a data structure in a way that retains most of the variation in it. The values for each gene are shown on the top panel (**a**), colored according to their assessed kinetic categories, while the values of the samples are shown on the bottom panel (**b**), colored according to hours past infection (hpi).

**Figure 8:**
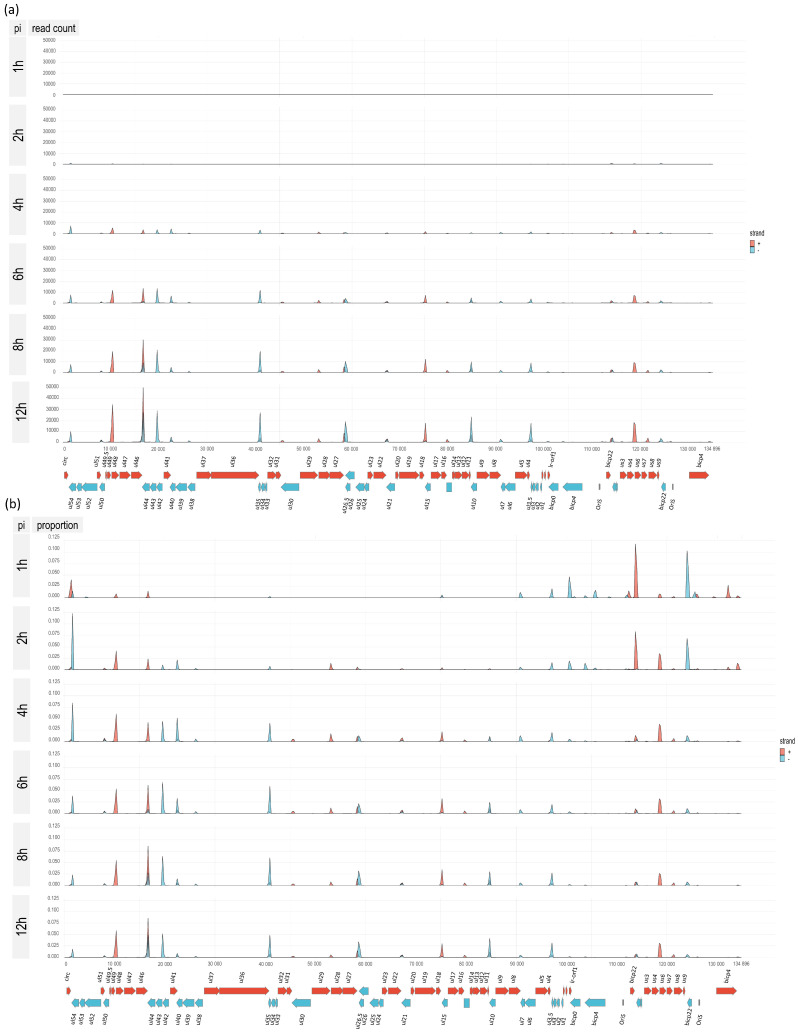
Genome-wide expression of transcription end sites. This figure shows the TES distribution along the viral genome in a time-varying manner using a density plot. (**a**) The *Y*-axis shows the number of TESs using the same scales. Red color indicates left-to-right orientation of the TESs, whereas blue color indicates the opposite orientation of the TESs. (**b**) This panel shows the proportion of the TESs within the total pool of TESs. Due to the use of common poly(A) signal by the members of tandem gene clusters, in most cases, we are unable to correspond the TSSs and the genes.

MinION data were base-called using Guppy base caller v. 6.1.4. with default parameters (--*qscore_filtering* = 9). The resulting barcoded reads were then mapped to the BoHV-1 genome (NCBI nucleotide accession: JX898220.1) using *minimap2* software [56], with the following parameters: -ax splice -Y -C5 --MD -un -G 5000 -g 1000. The ‘sam’ files resulting from the mappings were analyzed by the LoRTIA software (LoRTIA arguments) for adapter content, read orientation, for TSS, TES and intron content, and finally for transcript annotation. *IGV 2.7.2* was used to visualize transcripts and to generate Figure 1 and Figure S1. The transcripts were quantized in the samples from those reads that spanned from an annotated TSS to an annotated TES (as in [6,57]). The ‘*stranded_only*’ output of the LoRTIA analysis (containing only reads whose orientation could be assessed from the presence of either 3′ or 5′ adapters) was used to quantize TSS abundances, with a custom *R* script employing *rsamtools* [58] and *tidygenomics* (https://github.com/const-ae/tidygenomics, 12 August 2019). The reads were assigned to a canonical or alternative TSS based on the rules described in the Transcription start sites part of the Results section. The regions for each TSS, for which these reads were counted, can be found in the Table S1. The thus acquired gene/TSS abundances were used as an input for visualization with *ggplot* from the *tidyverse* [59] and *gggenes* (https://cran.r-project.org/package=gggenes, 24 June 2019) in Figures 2–8 and in Figures S2–S10. The counts were normalized to relative abundances by dividing the counts with either the total viral or host read count (assessed with *Rsamtools idxstats*). The heatmap in Figure 6 was generated using *complexheatmap* [60], while the PCA in Figure 7 was carried out using *factoextra* (https://cran.r-project.org/package=factoextra, 1 April 2020). *Isoformswitchanalyzer* [61] was employed to detect isoform-switches and their potential biological consequence, using the transcripts generated and LoRTIA software (https://github.com/zsolt-balazs/LoRTIA, 20 August 2019). We compared the 4, 6, 8, and 12 hpi samples to the 2 hpi samples using *dexseq* [61] as the read count was very low in the 1 hpi samples. The biological consequences of these switches were analyzed based on the presence/absence of protein domains of the *Pfam* database [62], the presence/absence of signal peptides with *SignalP* program (version 5.0) [63], and that of IDRs (intrinsically disordered proteins/protein regions) using *IUPred2A* [64].

## 3. Results

### 3.1. Time Course Analysis of BoHV-1 Transcriptome Using Nanopore Sequencing

In this work, we carried out a time varying in-depth analysis of the poly(A) fraction of the BoHV-1 lytic transcriptome over a 12-h period of productive infection on MDBK cells, using three biological replicates for each time point. An LRS technique based on the ONT MinION platform was employed for the analysis. We used non-amplified, direct cDNA sequencing for the library preparation to avoid the generation of spurious products by PCR. A frequent problem with PCR-based methods is that the production of amplicons is biased toward short fragment lengths, which results in an underestimation of the number of longer transcripts. Furthermore, direct cDNA sequencing circumvents the loss of sequences at the 5′-UTR of transcripts, which is a typical shortcoming of direct RNA sequencing. We used the ‘stranded only’ output of the LoRTIA program, containing every read whose orientation could be determined (based on the presence of either 3′- or 5′-adapters, or both). The applied conditions were indicated in every analysis.

### 3.2. BoHV-1 Expresses Four Immediate-Early Genes

MDBK cells were treated with 20 or 100 mg/mL cycloheximide (CHX), a protein synthesis inhibitor, prior to viral infection. Samples taken at 6 h and 8 h post infection (p.i.) were sequenced by the ONT dcDNA-Seq technique. We detected significant expression levels from the following five BoHV-1 genes: *bicp4*, *bicp22*, *bicp0, circ*, and *ul54* (Table 2). The bICP22 and bICP4 transcripts were expressed in very high levels in CHX-treated samples, whereas the other three genes produced a considerably lower number of transcripts. While, the *bicp22* gene was highly expressed throughout the entire examination period in untreated cells, the *bicp4* gene produced a low amount of RNA molecules (Table 2). We assume that despite its expression in CHX-treated cells, the BoHV-1 *ul54* is not a true IE gene because, unlike *bicp4*, *bicp22*, *bicp0*, and *circ*, whose expression levels were essentially unaffected by CHX treatment, *ul54* expression was drastically reduced by the increased CHX dose (see Discussion for explanation).

In this part of our work, we combined the data obtained in the CHX-treatment experiments and in our previous study [6] for identifying novel transcripts from the genomic regions of the BoHV-1 IE genes. As a result, we discovered even more complex transcript architectures (Figure 1, Table S2) than earlier, especially in the case of the transcripts of *bicp4* and *bicp22* genes. It can be seen in Figure 1 that a significant proportion of transcripts have alternative TSSs. We obtained few copies of full-length canonical bICP4 mRNA, since the amount of long transcripts is significantly underestimated by the applied sequencing technique. The *bicp4* gene produced several large-abundance TSS isoforms initiated from both the 5′-UTR and ORF region of this gene. Similarly, multiple TES isoforms were also observed, which terminated at both the 3′-UTR and the ORF of *bicp4* gene. This latter feature is very rare because the prototype herpesvirus transcripts do not tend to show a variance in TESs. Non-coding RNAs overlapping the long 5′-UTR and the long 3′-UTR of *bicp4* were also detected. The *bicp22* gene produces a large variety of transcripts with distinct splicing patterns and TSS polymorphism. This gene also codes for very long RNA molecules, which span a large part of the unique short (US) region of the genome. Moreover, we detected low abundance antisense RNA expression from the *bicp22* genomic region. The short TSS variants of the *bicp22* gene contain an upstream ORF (uORF) with a potential coding function [40], whereas the long canonical TSS isoforms have two additional uORFs in their 5′-UTRs (data not shown).

We detected high-abundance chimeric transcripts that contain the 5′-UTR region of *bicp4* gene and the entire *bicp0* gene, but the coding part of the *bicp4* gene is spliced out (Figure 1b), which is a confirmation of a previous report [65]. Interestingly, when examining bICP4′s second gene copy we found similar chimeric transcripts composed of the 5′-UTR of *bicp4* and the entire *circ* gene. These chimeric bICP4-circ transcripts span the genomic boundaries of the circular viral DNA and contain a large intron encompassing the *bicp4* ORF. Bicistronic ICP4-CIRC transcripts containing the entire *bicp4* and *circ* genes were also detected. It is unknown whether they are unprocessed or mature transcripts. We propose that the IE expression characteristic of the BoHV-1 *circ* gene is afforded by the *bicp4* promoter, the IE transcription unit 1 (IEtu1) [65]. The TAATGAGCT sequence of the *bicp4* promoter has been described as the binding site for the tegument VP16 transactivator [63]. We also detected a similar sequence (TAATCGAGA) within the distal promoter of *bicp22* gene, or IEtu2 [66]. No TAATGARAT-like sequences were detected in the promoter of the other three IE genes. Therefore, of the four IE genes, *bICP0, bICP4* and *circ* appear to be controlled by IETu1, while *bICP22* is controlled by IETu2.

The replication origin (OriS) of BoHV-1 is overlapped with two oppositely oriented (sense/antisense) IE transcripts. One of these replication origin-associated RNAs (raRNAs) is a very long bICP4 TSS transcript isoform, whereas the other one is an oppositely oriented, overlapping an also very long TSS variant of the bICP22 transcript. Their abundance is likely underestimated because both transcripts are very long. The *bicp22* promoter IETu2 containing the TAATCGAGA sequences and the associated TSS overlaps the OriS.

### 3.3. Transcription *Start Sites*

In our previous work, we have identified a large number of novel BoHV-1 TSSs with the help of LoRTIA software [6]. In this present work, the BAM files processed by LoRTIA suite were used to assign each read to a specific, annotated TSS. Besides confirming all previously detected TSSs, we identified a few novel alternative TSSs (aTSS) that were filtered out earlier from the transcript annotation pipeline (Table S1). A TSS is termed alternative if its distance from the canonical TSS is at least 50 bps, and its ratios reach at least 5% of the canonical TSSs encoded by the same gene. A TSS represented in less than 5% is termed as rare TSS. The rules for the assignment of the reads to TSSs were as follows. First, we evaluated for each gene to determine if they produce alternative TSSs or not. Then, the 5′-ends of the reads were assigned to the genes differently, depending on whether the gene produced alternative TSSs or not. If a gene didn’t produce alternative TSSs, all reads with 5′-ends downstream from the canonical TSS were assigned to the gene. However, if the gene did produce alternative TSS(s), then only those reads whose 5′-ends were at most 200 bps downstream from it were assigned to the canonical TSS, while reads were assigned to the alternative TSSs if their 5′-ends were in a +/−10 bp window. This interval was used to eliminate the effect of the possible degradation of reads from the 5′-end, incomplete RT or sequencing artifacts. In the case of polycistronic transcripts, the rules were applied as if the downstream genes would be alternative TSSs of the canonical TSS of the most upstream gene. That is, reads were assigned to the most upstream gene in a −10/+200 bp window, while in the case of the downstream genes in a +/−10 bp window. These rules were applied because with the exception of the TSSs located within the monocistronic genes, it is not possible to ascertain whether the alternative TSSs are the transcription initiation sites for 5′-truncated genes or for long TSS isoform of the downstream genes in a tandem gene cluster.

In this part of the study, we generated quantitative maps of TSSs at a single-nucleotide resolution for BoHV-1. Figure 2 shows the TSSs in the selected genes (a, b: *bicp22*; c, d: *ul26.5-26*; e, f: *ul10*). The *bicp22* gene is regulated in a complex manner in BoHV-1 and also in other alphaherpesviruses. It can be seen that besides the canonical TSS (the most distal TSS), an alternative TSS is also located downstream of it, and there are also additional low-abundance TSSs. Rare TSSs were also identified outside and within the ORF of this gene. Figure S2 shows that non-stringent filtering can produce a few false transcripts, however, the majority of them are likely true transcripts with imperfect adapter sequences. Even stringent filtering allows the appearance of very low abundance TSSs, which may be artifactual, but it is also possible that transcription initiation may occur randomly throughout the entire genome with a low incidence. Alternative TSSs can also be located within the ORF in an in-frame position, and potentially code for an N-terminal-truncated polypeptide. Such is the case of the *ul26* gene, which encodes the *ul26.5* embedded gene (Figure 2C,D). When changing the scale in the y axis (panels B, D, and F), low-abundance TSSs are visible. The *ul10* gene expresses transcripts from at least three promoters, generating two additional TSSs besides the canonical TSS, one of which is located within the ORF and the other one at an upstream position within the adjacent *ul9* gene encoded in the reverse strand. Figure S2 shows that false TSSs are produced if only the presence of the 3′-adapters on the reads was the requirement for the annotation of a TSS.

### 3.4. Transcription Start Site Clusters

Our work demonstrates that viral promoters are multimodal, that is, they tend to generate closely spaced TSSs instead of specifying a single transcription initiation site. This phenomenon is conceptually different from those of alternative promoters, which are separated by considerably longer genomic regions. We define a canonical TSS cluster (TSSC) as those TSSs that include the most abundant canonical TSS and the TSSs in its vicinity (within ±35 bps). The same definition is applied to the alternative TSSCs. We found that the distributions of TSSs exhibit distinct patterns in different genes, but similar arrangements of the same gene at different time points (Figure 3). Generally, a dominant TSS is surrounded by high-abundance TSSs, but alternative TSSs and a relatively large number of rare TSSs are also encoded at larger distances within the given gene or in the upstream genes. However, we cannot exclude that many, if not all, the rare TSSs are technical and not biological products.

### 3.5. Time-Course Genome-Wide Expression of Transcription Start Sites

In this part of the study, we examined the time-varying distribution of the TSSs of BoHV-1 transcripts. Figure 4 shows the expression of TSSs along the entire viral genome using a density plot, whereas Figure S8 Panels a–c show the same using bar plot illustrations in the same (a), or in higher resolution [maximum 5000 (b), or 500 (c) TSSs are allowed in a single base] during the course of infection. By definition, alternative

TSSs are expressed in a lower amount than canonical TSSs, which is not always manifested in the obtained TSS abundance of long transcripts due to the negative bias of LRS towards long reads.

Next, we analyzed the transcriptional kinetics of the canonical and the high-abundance alternative BoHV-1 TSSs throughout the course of viral infection (Figure 5 and Figure 6; Supplementary Figures S4–S6). As described above, only the reads from the LoRTIA output were used to ensure the exclusion of technical artifacts and spurious transcripts. The count data for the comparative kinetic analysis was obtained by summing the 5′-ends of reads that could be assigned to either a canonic or an alternative TSS, but these were counted separately. The expression values for each TSS were calculated by dividing their read counts with the total viral read count. Then, we classified the genes on the basis of these expression kinetics, i.e., the shape of their expression curves (Figure 5). This categorization was carried out exclusively by the analysis of the transcription dynamics of the RNA molecules and the results were compared to previously described results, obtained by conventional methods [67,68,69,70,71]. We considered a transcript as IE if it reached its maximal level at either 1 hpi or 2 hpi, but in the latter case this transcript also had to be expressed at high level at 1 hpi. The E transcripts were defined as having their maximum expression at 2 or 4 hpi, but in the former case the 1 hpi expression level had to be relatively low. According to this classification, the *ul54* gene is an E gene, which further confirms our conjecture that it may not be a true IE gene. E/L transcripts have their maximum at 6 hpi, whereas L transcripts have their peaks at either 8 or 12 hpi. This categorization revealed the canonical promoter of *circ* gene and that both the canonical and the strongest alternative promoter (controlling the expression of bICP22-S2 transcript) exhibit an IE kinetics although they do not contain TAATGARAT-like sequences. Thus, the IE characteristics of these two genes are not solely determined by the *icp4* promoter, as discussed above. Several very-low-abundance transcripts showed relatively high standard deviations at certain time points or along the entire examination period in certain transcripts, which makes their kinetic characterization unreliable.

Figure 7 shows a Principal Component Analysis (PCA) of the normalized gene counts. We used PCA to visualize overall trends, differences, and similarities between the gene expressions in different samples. The result shows that the triplicates clearly cluster together based on their *hpi* categories, which supports that the within-sample variation is lower than the between-sample variation and that no samples seem to be outliers. Moreover, a definite trend can be observed from the 2 hpi samples to the 12 hpi samples, showing that the change in the overall expression progresses in accordance with sampling time.

We also characterized the transcript dynamics by using the total host reads as a reference (Figure S3). These curves do not exhibit such marked differences between the time points as when they were normalized to the total viral read counts. The reason for this is that the copy number of the DNAs increases with time and therefore, the levels of gene expressions are also globally increased. Using the host reads as reference yields less meaningful data since viral infection leads to the degradation of host transcripts, the extent of which increases over time. Furthermore, this latter method is prone to sampling bias, which mainly originates from the varying virus/cell ratio used for infection. In order to mitigate these distorting effects, we characterized the transcription dynamics by calculating the difference (Figure S6) or the ratio (Figure S7) of the relative proportions of the transcripts (normalized to total host reads) within two consecutive time points (delta values and fold values, respectively). The different ways of characterization allow a more precise analysis of the gene expressions.

### 3.6. Transcription End Sites

Alphaherpesviruses have a far higher level of variety in their TSSs than in the TESs, and only a few genes have alternative TESs. Transcription end sites can also form clusters: canonical TESs are surrounded by large abundance TESs in their immediate vicinity (Figure S8). The existence of transcript end-site clusters (TESCs) implies that the biological mechanisms behind transcription termination is not precise at the nucleotide-level. The dynamics of genome-wide TES expression is illustrated in Figure 8 and Figure S9.

### 3.7. Genome-Wide Expression Dynamics of BoHV-1 Transcripts

**Figure 9:**
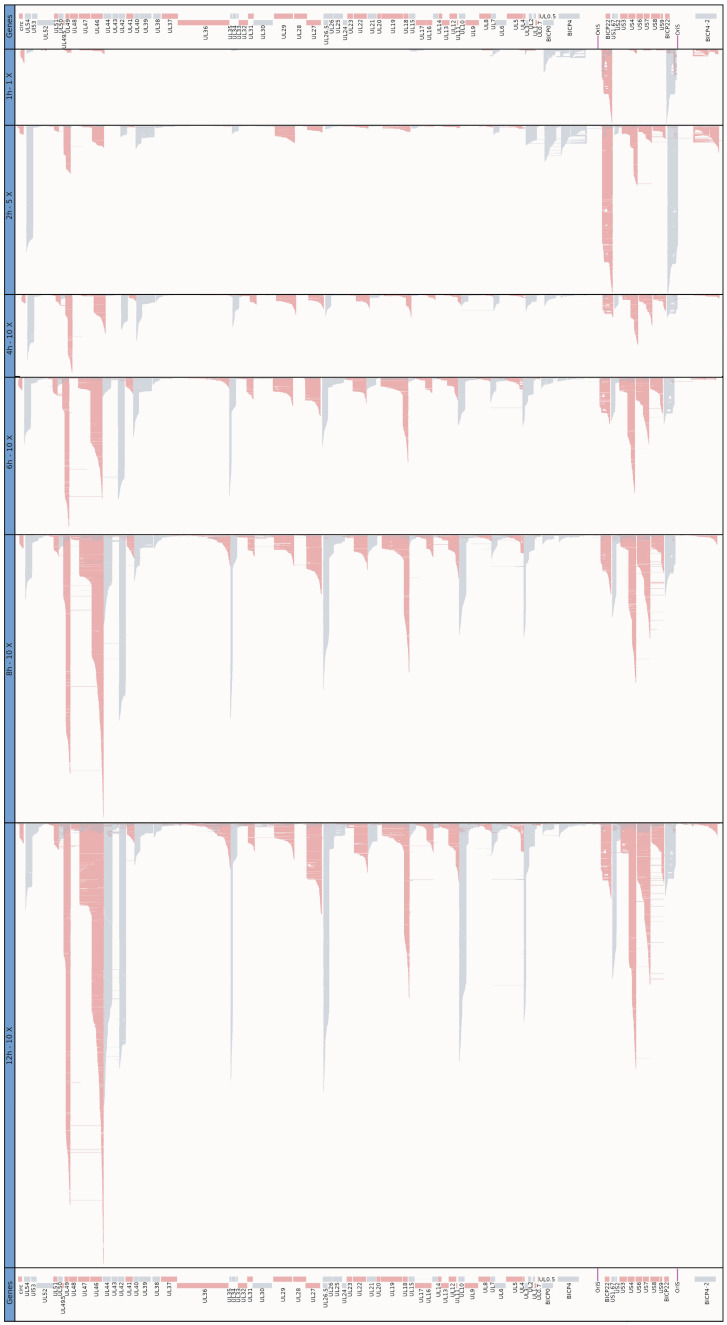
Genome-wide expression dynamics of BoHV-1 transcription. Viral transcripts were analyzed at six time points (1, 2, 4, 6, 8, and 12 h) throughout the viral infection. The average of the three biological replicates was depicted using *IGV 2.7.2*. Red color indicates a left to right (+ strand), whereas gray color a right to left orientation (− strand) of genes and transcripts. The 1 h sample was not down-sampled, while at the 2 h sample, we applied a twofold and at the other time points a tenfold down-sampling.

We used the LoRTIA transcripts for the illustration of global gene expression in Figure 9 and Figure S10. At the first hour of infection, we can observe a relatively high expression level of IE genes, especially of *bicp22*, *bicp0*, and *bicp4* genes. The number of long transcripts is underestimated due to the size bias of the sequencing technique. At 2 hpi, the *bicp22* and *ul54* genes are highly upregulated, while at 4 hpi, besides the *ul54*, the *ul49.5-49* and *us3-4* genomic regions are especially transcriptionally active. These latter two regions remained highly active during the entire examination period. Later on, the *ul46-47* region exhibits the highest expression level. This picture clearly illustrates the temporal changes of the transcript ratios within a tandem gene cluster. In these clusters, first the longer, polycistronic RNAs appear to be expressed at a high proportion, which is followed by the increase in the ratio of shorter, downstream transcripts.

However, since there are some exceptions, this cannot be considered as a general rule. Figure 9 also shows that besides the canonical transcripts, many RNA isoforms and truncated transcripts are expressed at a high level and ratio. There is a highly complex, temporarily changing patterns of transcriptional overlaps formed by divergent, parallel, and convergent gene pairs, or by transcriptional read-throughs of tandem and convergent genes. Transcriptional overlaps can also be formed via transcriptional read-through from distal genes, which thereby produce complex transcripts containing at least one gene with opposite orientation relative to the other genes. Additionally, transcriptional overlaps can be formed by antisense RNAs produced by their own promoters, such as those ones overlapping *bICP22*. It can be seen in Figure S1, generated using LoRTIA, that every divergent gene pair produces overlapping transcripts. Due to the stringent criteria applied for the annotation, many long transcripts were lost, which led to a significant reduction of overlapping complexity. Tandem genes use two ways for the generation of transcriptional overlaps: through transcriptional read-throughs across the downstream gene(s) by the upstream gene(s) and through forming tail-to-head overlaps by the upstream and downstream partner genes.

### 3.8. Time-Dependent Expression of Viral Gene Domains

Transcript isoforms (especially of splice variants) carrying distinct protein domains are frequent in human cells and were shown to be important in cancer formation [72]. In viruses, where the complexity of transcripts and the number of transcript isoforms expressed from the same gene is much higher, this phenomenon may be even more important. The changes in transcript isoform ratios can induce different functionality, i.e., biological consequences via altering the protein domains of the coding sequences, as well as via changing the presence/absence of their signal peptides and IDRs (intrinsically disordered protein regions). To examine these possible phenomena in the BoHV-1 transcriptome during viral infection, we used the *isoformswitchanalyser* tool [72] on the LoRTIA transcript counts.

**Figure 10:**
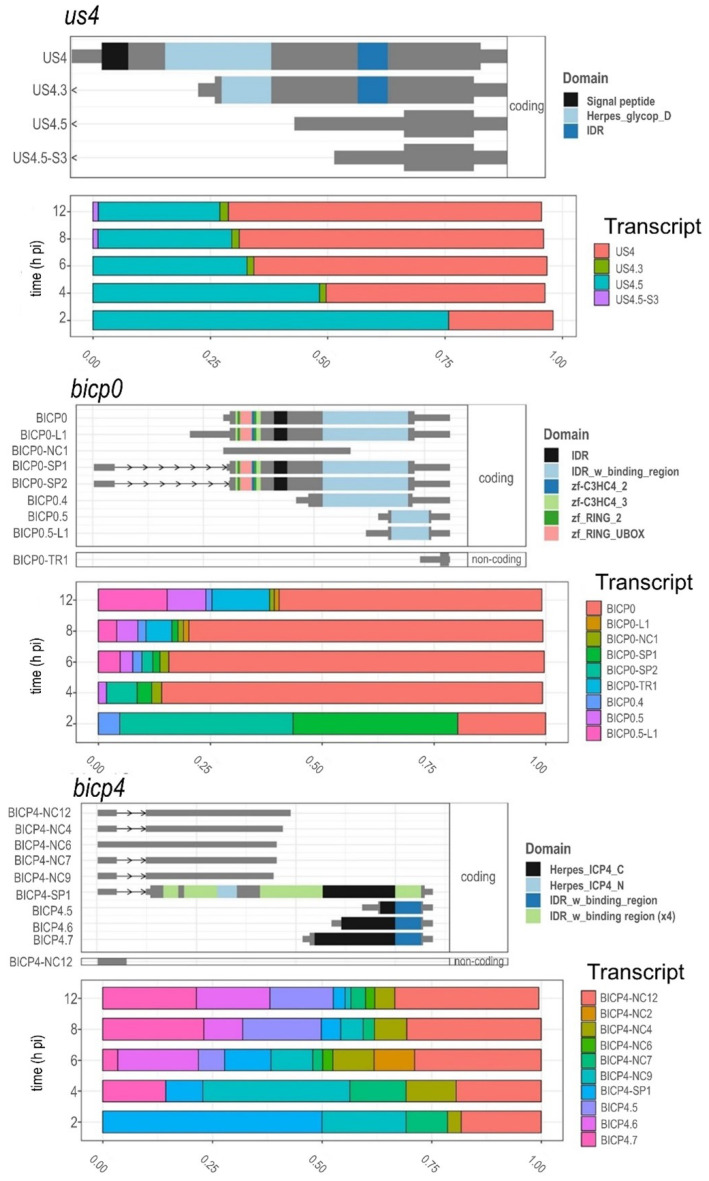
The colored boxes show the protein domains identified from the Pfam database. Isoform structure and expression profile of *bicp4*, *bicp0*, and *us4* genes during the viral infection. An isoform switch was detected in these genes as the result of an altered transcript expression profile, and moreover, these switches induced consequences as well, as is a change in their protein domain structure, signal peptide content or predicted protein disorder (IDR, intrinsically disordered proteins/protein regions). The transcript isoforms are faceted according to their coding probability (assessed with CPAT2 REF). The 3′- and 5′-truncated transcripts contain only predicted ORFs.

Figure 10 shows the annotated protein domains, signal peptides, and IDRs of the transcripts of selected genes. The transcripts were filtered to a cutoff of 0.001 (only those transcripts were kept that reached 0.1% of their parent gene’s abundance in any of the samples). The results of this analysis showed that the 5′-truncated transcripts of the *us4.5* gene lacks all the identified protein domains and the signal peptide compared to the canonical US4 transcript. The data showed that initially the *us4.5* gene products are generated in a higher proportion, but at the later phase of infection, the US4 transcripts of the *us4* gene became dominant (Figure 10). This might mean that after an initial ‘noisy’ transcription leading to transcripts without functionality, the transcription switches to express functional mRNAs. This observation is supported by the fact that *us4* is a late gene, which is not required in the early stage of infection. We cannot exclude that the US4.5 transcript is functional, and it is possible that it might regulate the expression of *us4*. A similar pattern was found in the case of the *ul44* gene: in the 4 hpi samples there were many truncated isoforms expressed along with the canonic UL44, but by 6 hpi these were all reduced significantly, and the expression profile settled to the canonic isoform.

In the case of the immediate early *bicp4* gene, this was quite the opposite: the canonical bICP4-SP1 transcript was dominant in the 2 hpi sample only. In the 4 hpi samples the 3′-truncated transcripts [named NC (non-coding) due to the lack of in-frame stop codon] were expressed in large abundance; but afterwards, starting in the 6 hpi samples, they started to decrease favoring the 5′-truncated transcripts (bICP4.5, 4.6 and 4.7). By the 8 and 12 hpi samples, *bcip4* transcripts, which do not carry the C-terminal part of the viral ICP4 protein domain (Herpes_ICP4_C), became the most abundantly expressed *bicp4* isoform.

The *bicp0* gene expresses two splice variants (with the same domain structure) along with the canonic isoform in approximately equal amounts, but after 2 hpi a significant decrease was observed. Several 5′-truncated isoforms (without several annotated domains) were expressed in minor abundances in the later stages of the viral infection. Several other genes were also identified by *isoformswitchanalyser* to show isoform switching with a ‘consequence’ (*ul18, ul21, ul40, ul44*), but in these genes the isoform ratio was very similar in every sample after the 2 hpi samples (Figure S11).

## 4. Discussion

The last couple of years has witnessed a significant advance in long-read sequencing technologies. High-throughput LRS methods are able to determine full-length RNA molecules, which allow us to disclose a more intricate picture of the transcriptomes compared to the former methods. These investigations have demonstrated that herpesviruses exhibit astoundingly complex transcription profiles [37,41,73]. LRS is able to distinguish between co-oriented overlapping RNA molecules and transcript isoforms, thereby allowing the kinetic characterization of transcripts that resisted this endeavor before.

Our previous analysis has identified a large number of novel transcripts and transcript isoforms of BoHV-1 [6]. Here, we report the analysis and categorization of the BoHV-1 dynamic transcriptome. We used direct cDNA sequencing because it produces longer reads than the amplification-based cDNA sequencing and—in contrast to direct RNA sequencing—it is able to read the complete 5′-ends of the leader regions of transcripts. At the same time, direct cDNA sequencing lacks the false transcripts generated by PCR, and also the amplification biases leading to improper quantitation [74,75].

Recent evidence has shown that—in contrast to the conventional view—promoters are structurally complex and generate a significant TSS polymorphism in cellular organisms [76,77,78,79]. Our investigations identified a fine-scale regulation of transcription initiation events at the base pair level in the BoHV-1 genome. Alternative TSSs are controlled by separate promoters. The importance of non-canonical TSSs has long been underestimated; they may be part of the complex regulatory code of the gene expression. We detected two novel uORFs in the 5′-UTR of the longer TSS isoform of bICP22 transcript, which might be translated. It has been demonstrated that these uORFs have a significant role in gene-expression regulation [40]. The differential use of these uORFs in transcripts encoded by the same gene may have an important role in the molecular pathogenesis of viruses. We found that besides canonical transcription end sites, several other large abundance TESs are located in its immediate vicinity, which indicates that the mechanism of the transcription termination is not precise to the nucleotide level and is also temporally regulated.

LRS methods are able to differentiate between monocistronic and multicistronic transcripts. This is important when assessing the kinetics of the proteome from the transcriptomic data because only the most upstream CDS is translated from multicistronic RNA molecules. LRS also allows us to distinguish between the potentially many transcript isoforms produced by a single gene. Since in most cases the canonical transcripts are by far the most abundant, the total and canonical transcript dynamics are similar to each other. However, genes with complex gene-expression regulation are important exceptions to this general pattern.

We characterized the transcripts according to their transcription dynamics throughout the course of infection and compared these results with those that used DNA polymerase inhibitors for distinguishing between early and late genes. We applied a translation inhibitor (cycloheximide) to identify the immediate–early genes. We identified four genes with immediate–early expression: *icp4*, *icp22*, *icp0*, and *circ*. While circ is often not cited as an immediate–early gene in most papers today, it was shown in an early study to have this kinetic class [13]. We found that the *icp4* promoter and the promoter controlling a long transcript isoform of *icp22* contain TAATGARAT-like sequences that bind to the tegument VP16 protein with the help of cellular factor Oct-1 [7], to assist in IE transcription. In addition, we found that the immediate-early kinetics of *circ* expression is at least partly controlled by the utilization of the *icp4* promoter. The evidence of this is the existence of the chimeric ICP4/CIRC transcript. Such is the case of bICP0 and bICP4, which are transcribed from the IE transcription unit 1 as a single transcript that undergoes two different splicing events, generating bICP4 RNA (IER4.2) and bICP0 RNA (IER2.9) [65]. We could not identify TAATGARAT-like sequences in the *ul54* promoter. We cannot exclude the possibility that *ul54* is an IE gene in BoHV-1, but other observations also suggest that this is probably not the case. The expression of *ul54* gene in CHX-treated cells can be explained by the fact that at the early stage of infection this gene is expressed in the highest level, which can also be seen at suboptimal (20 mg/mL) CHX doses (Table 2). We can only speculate that the 100 mg/mL CHX concentration may have been insufficient for completely blocking the transcription of *ul54* gene, or conceivably the tegument bICP4 transactivator was able to induce the observed low-level expression from this gene (BoHV-1 bICP4 transactivator has also been shown to be present in the viral tegument [80]). Pokhriyal and colleagues [81] have reported the identification of three additional IE genes (*ul21*, *ul33*, and *ul34*), however, we could not confirm this result. We assume that the authors might have applied inadequate amount of CHX for the infected cells.

Additionally, the *ul54* is an IE gene in the *Simplexvirus* genus, but not in the *Varicellovirus* genus of alphaherpesviruses to which the BoHV-1 belongs, where this is an early gene.

While the location of OriS appears to be conserved in all alphaherpesviruses (upstream of the *ul1* gene), OriL is missing in VZV and BoHV-1, or maps to different genomic locations: between the *ul29* and *ul30* genes in HSV-1 and between the *ul21* and *ul22* genes in PRV and equid herpesvirus 1 (EHV-1) [82]. In this work, we demonstrated that the very long 5′-UTR isoforms of the bICP4 and bICP22 transcripts overlap the OriS and also each other. This type of organization of raRNAs has been described in HSV-1, but in other alphaherpesviruses, such as pseudorabies virus and varicella-zoster virus, only the long 5′-UTR isoform of bICP22 homolog has been detected [83]. However, as the 5′-UTR-bICP4 isoform is a very long RNA molecule, its homologues might exist but have gone undetected in other alphaherpesviruses. Furthermore, the *bicp22* promoter containing the TAATGARAT-like sequences and the associated TSS overlap with the OriS is unique among the alphaherpesviruses.

Our work explored a highly extensive transcriptional overlapping meshwork in BoHV-1. Transcriptional overlaps are generated by transcriptional read-throughs between tandem and convergent genes or by the shared utilization of genomic loci by divergently or parallelly oriented genes. We have previously shown that in the case of Herpes Simplex Virus (HSV-1), each convergent gene pair produced non-polyadenylated read-through RNAs overlapping the partner genes [41].

Convergent and divergent transcriptional overlaps generate antisense regions on the transcripts. Parallel overlaps produce multicistronic RNA molecules, such as ‘abcd’, ‘bcd’, and ‘cd’ transcripts, through a transcriptional read-through mechanism. Additionally, transcription from the downstream genes of a tandem cluster is always initiated within the upstream genes. The transcriptional overlaps cannot be solely explained by the economic organization of the viral genome. It has been proposed that they function as a transcriptional interference network, based on the interaction between the transcriptional machineries [84]. This proposed mechanism might represent a novel, genome-wide level of gene-expression regulation. Furthermore, the overlapping long TSS transcript variants of bICP4 and bICP22 might interfere not only with each other’s transcription but also with the DNA replication due to their overlap mutual of OriS [82]. Likewise, the co-localization of transcription and replication initiation, through the overlap of OriS with the IE promoter (containing VP16 binding site) of *icp22* gene, also suggests an interference between the two apparatuses. Possibly, the interaction is the main function of this promoter and not the generation of transcripts, which might explain their relatively low abundance. In this scenario, IE transcription from this b*icp22* promoter might inhibit DNA replication, whereas at later times in the viral cycle, DNA synthesis might exert a repressive effect on the transcription. This potential interplay between the two apparatuses might control the orientation of the progression of the replication fork.

## Figures and Tables

**Table 1:** Barcode sequences used for labeling the different samples.

	Time Point	Barcode	Barcode Sequence
1st replicate	1 h	A1(BC01)	AAGAAAGTTGTCGGTGTCTTTGTG
	2 h	A2(BC02)	TCGATTCCGTTTGTAGTCGTCTGT
	4 h	A3(BC03)	GAGTCTTGTGTCCCAGTTACCAGG
	6 h	A4(BC04)	TTCGGATTCTATCGTGTTTCCCTA
	8 h	A5(BC05)	CTTGTCCAGGGTTTGTGTAACCTT
	12 h	A6(BC06)	TTCTCGCAAAGGCAGAAAGTAGTC
	MOCK	A7(BC07)	GTGTTACCGTGGGAATGAATCCTT
2nd replicate	1 h	A8(BC08)	TTCAGGGAACAAACCAAGTTACGT
	2 h	A9(BC09)	AACTAGGCACAGCGAGTCTTGGTT
	4 h	A10(BC10)	AAGCGTTGAAACCTTTGTCCTCTC
	6 h	A11(BC11)	GTTTCATCTATCGGAGGGAATGGA
	8 h	A12(BC24)	GCATAGTTCTGCATGATGGGTTAG
	12 h	A1(BC01)	AAGAAAGTTGTCGGTGTCTTTGTG
	MOCK	A2(BC02)	TCGATTCCGTTTGTAGTCGTCTGT
3rd replicate	1 h	A3(BC03)	GAGTCTTGTGTCCCAGTTACCAGG
	2 h	A4(BC04)	TTCGGATTCTATCGTGTTTCCCTA
	4 h	A5(BC05)	CTTGTCCAGGGTTTGTGTAACCTT
	6 h	A6(BC06)	TTCTCGCAAAGGCAGAAAGTAGTC
	8 h	A7(BC07)	GTGTTACCGTGGGAATGAATCCTT
	12 h	A8(BC08)	TTCAGGGAACAAACCAAGTTACGT
	MOCK	A9(BC09)	AACTAGGCACAGCGAGTCTTGGTT

**Table 2:** Genes expressed in CHX-treated cells.

Gene	Sequence Names	Start	End	6 h 20 mg	8 h 20 mg	6 h 100 mg	8 h 100 mg
***bicp22***	JX898220.1	112,888	113,790	132,263	165,542	138,780	127,408
***bicp4***	JX898220.1	103,907	107,941	44,292	57,067	36,249	43,288
***circ***	JX898220.1	487	1227	5781	8667	6137	5398
***bicp0***	JX898220.1	100,898	102,949	4459	5270	4529	4234
***ul54***	JX898220.1	1648	2850	13,953	62,679	4601	4841

## Data Availability

The datasets used for this study are submitted to the European Nucleotide Archive’s SRA database and are available under the accession PRJEB33511.

## Supplementary Materials

The following supporting information can be downloaded at: https://www.mdpi.com/article/10.3390/v14061289/s1, Figure S1: Whole-genome transcript dynamics; Figure S2: Long-read sequencing produces false TSSs; Figure S3: Whole-genome Kinetics of TSSs illustrated by bar plot; Figure S4: The proportion of the viral and host transcripts throughout the infection; Figure S5: Expression kinetics of BoHV-1 transcripts; Figure S6: Expression kinetics of BoHV-1 transcripts—delta values; Figure S7: Expression kinetics of BoHV-1 transcripts—fold values; Figure S8: TES clusters; Figure S9: Dynamics of TES expression along the entire viral genome; Figure S10: Whole-genome transcript dynamics; Figure S11: Isoform-switching in ul44, ul18, ul21 and ul40 genes; Table S1: List of transcription start and end sites of BoHV-1 transcripts; Table S2: BoHV-1 IE transcripts.
