# Supplementary figures and images for: In-Depth Temporal Transcriptome Profiling of an Alphaherpesvirus Using Nanopore Sequencing
Source: Viruses. 2022 Jun 13;14(6):1289. doi: 10.3390/v14061289 (PMC9229804; doi:10.3390/v14061289)

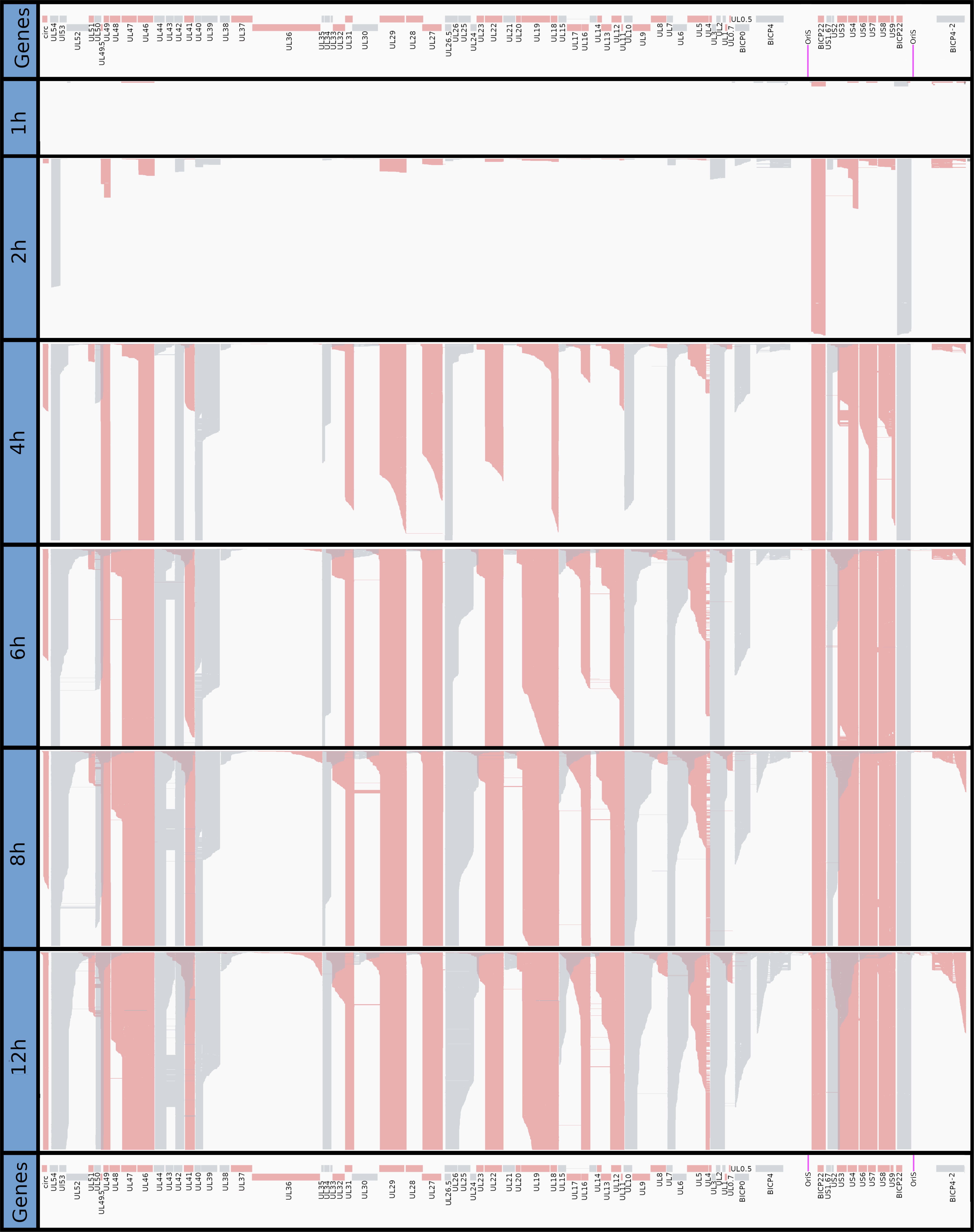

Supplement: Supplementary file 1 [file viruses-14-01289-s001.zip › SupplementaryFigureS1.tiff]

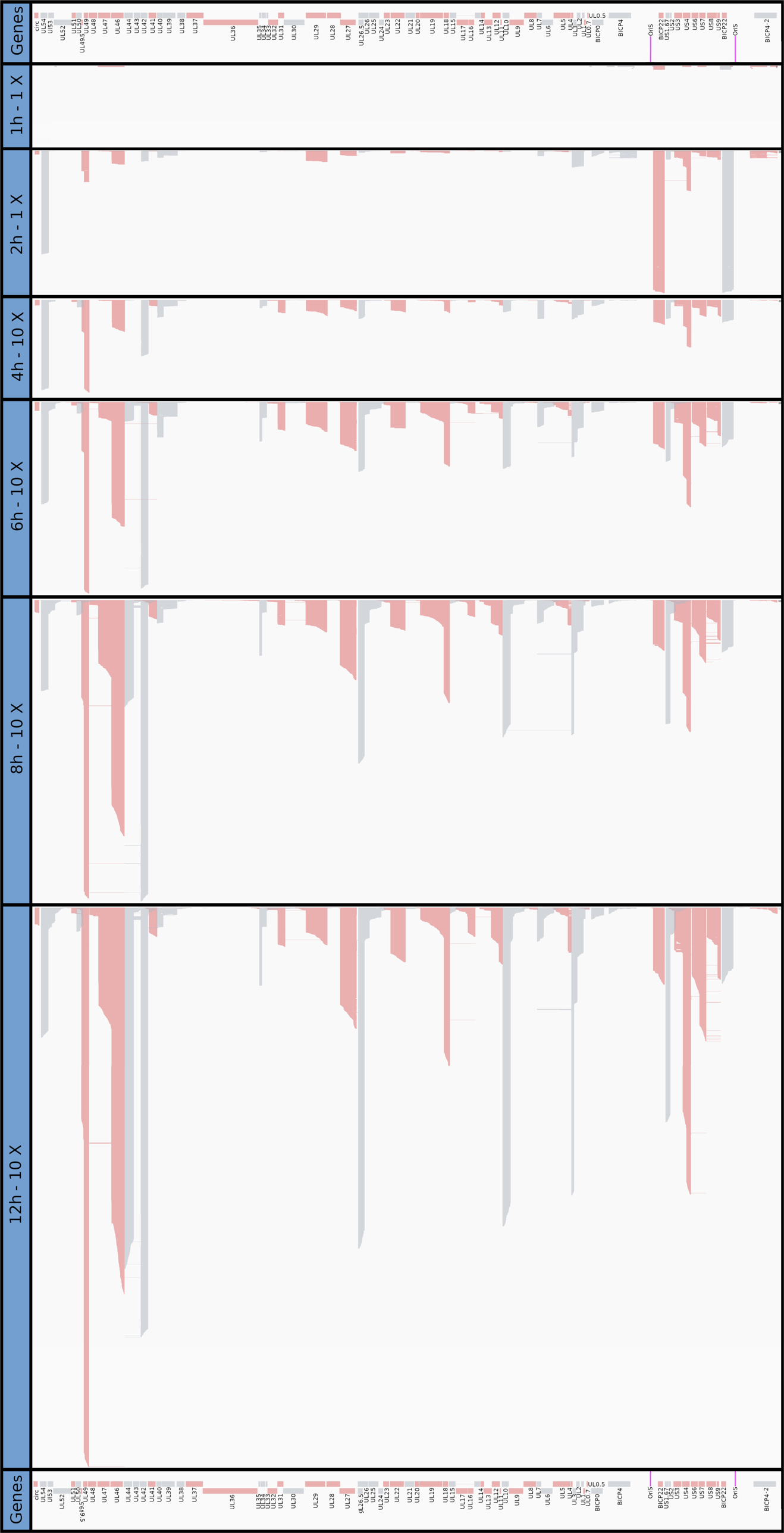

Supplement: Supplementary file 1 [file viruses-14-01289-s001.zip › SupplementaryFigureS10.tiff]

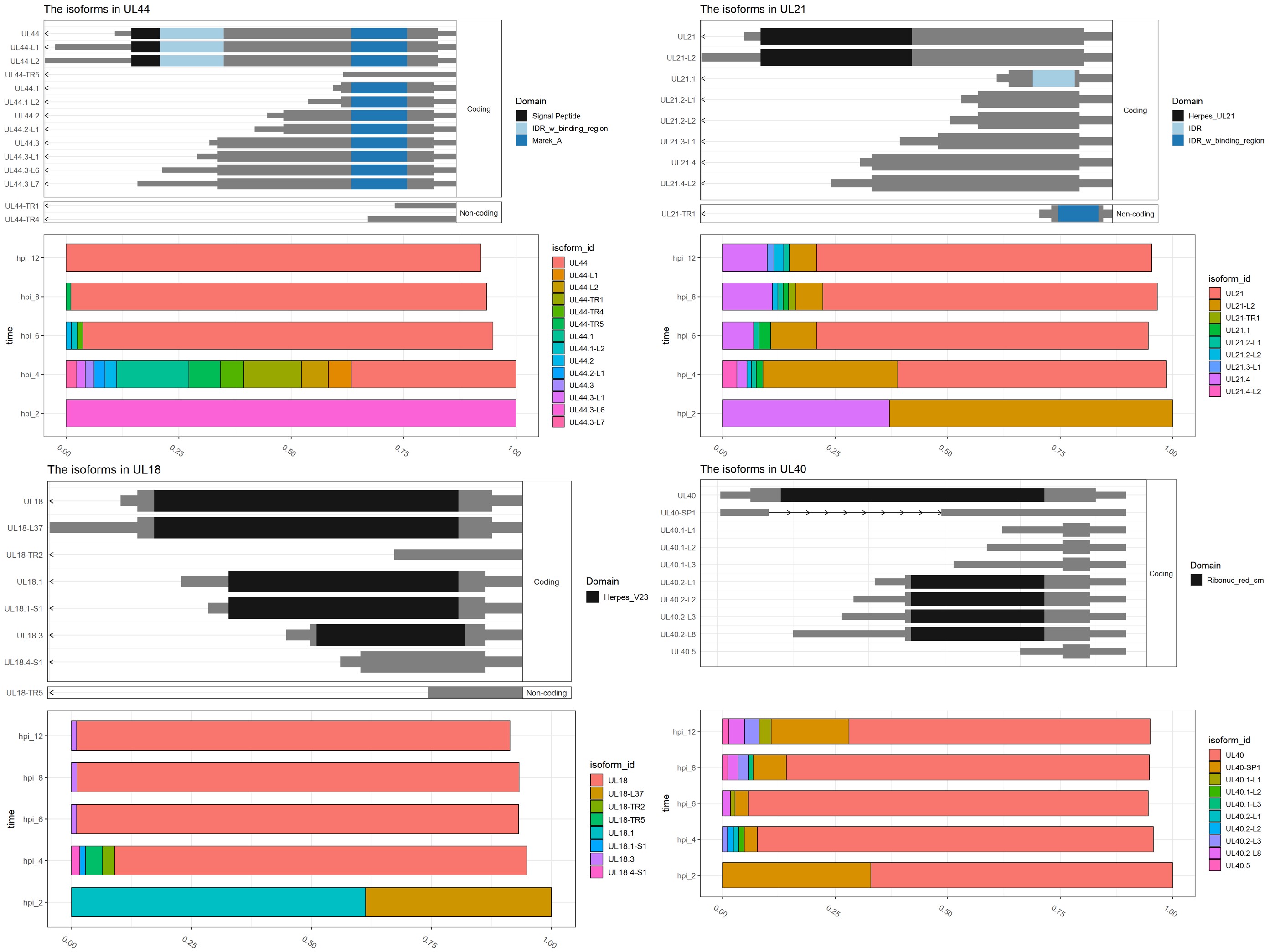

Supplement: Supplementary file 1 [file viruses-14-01289-s001.zip › SupplementaryFigureS11.jpg]

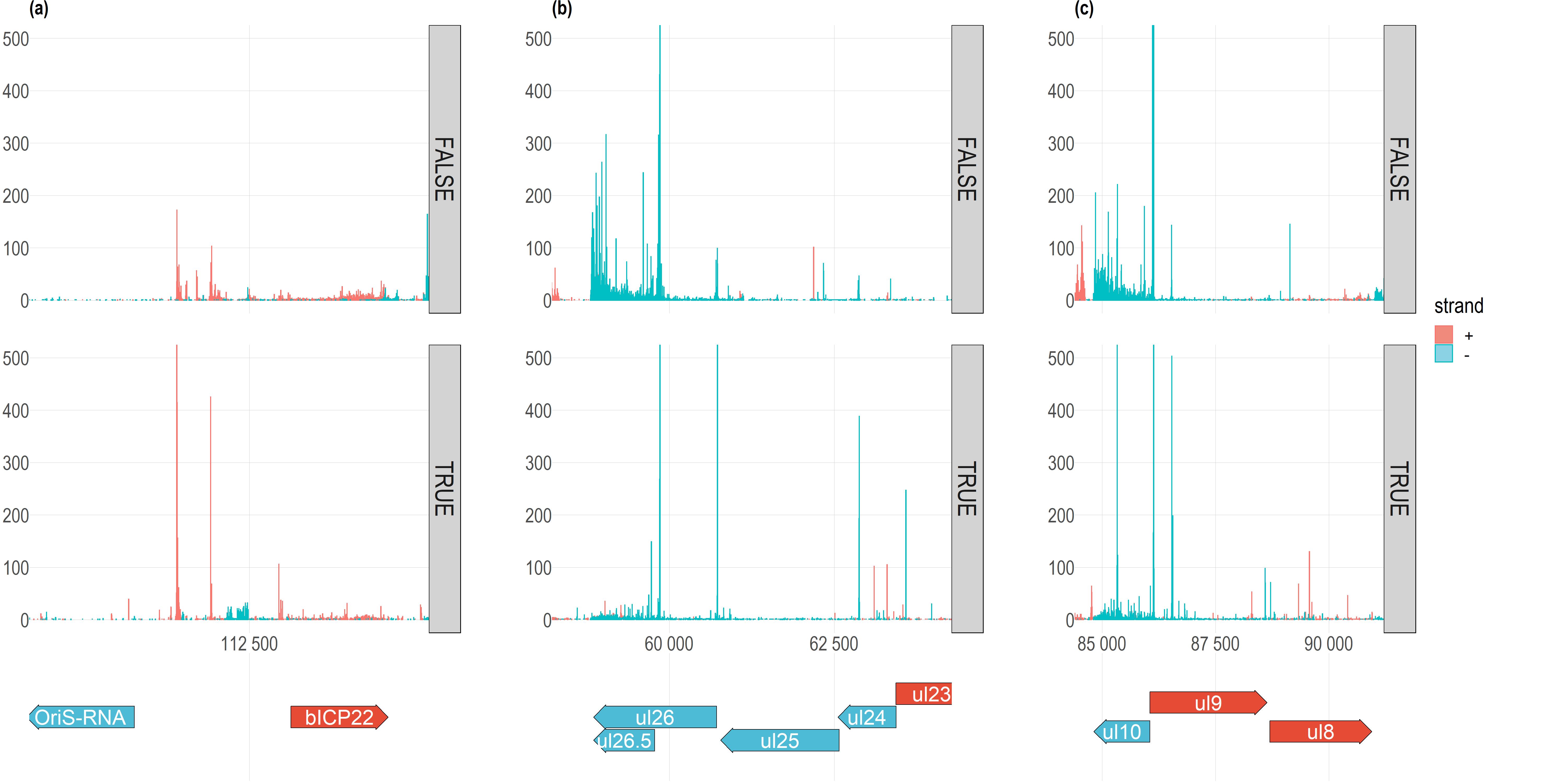

Supplement: Supplementary file 1 [file viruses-14-01289-s001.zip › SupplementaryFigureS2.jpg]

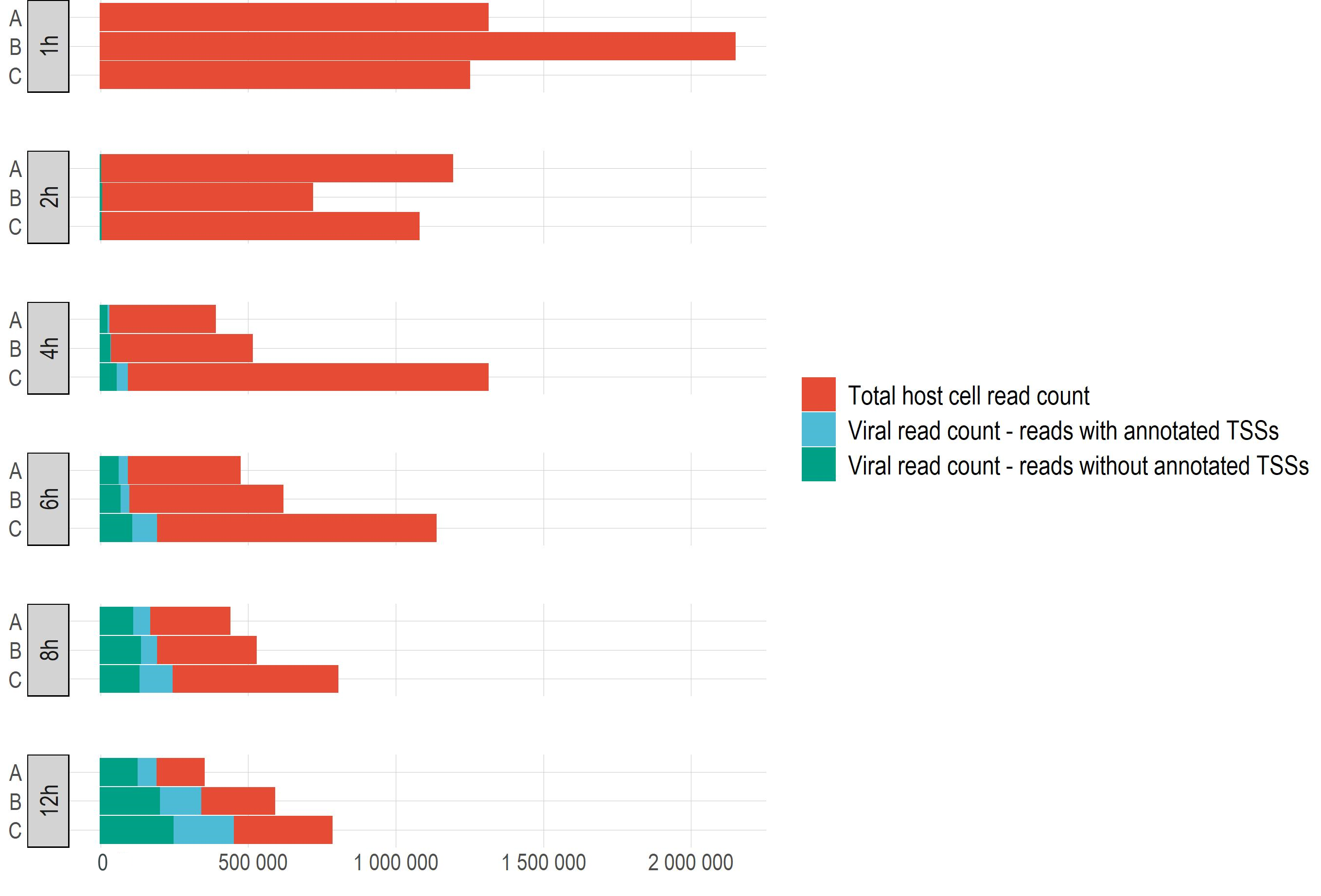

Supplement: Supplementary file 1 [file viruses-14-01289-s001.zip › SupplementaryFigureS4.tif]

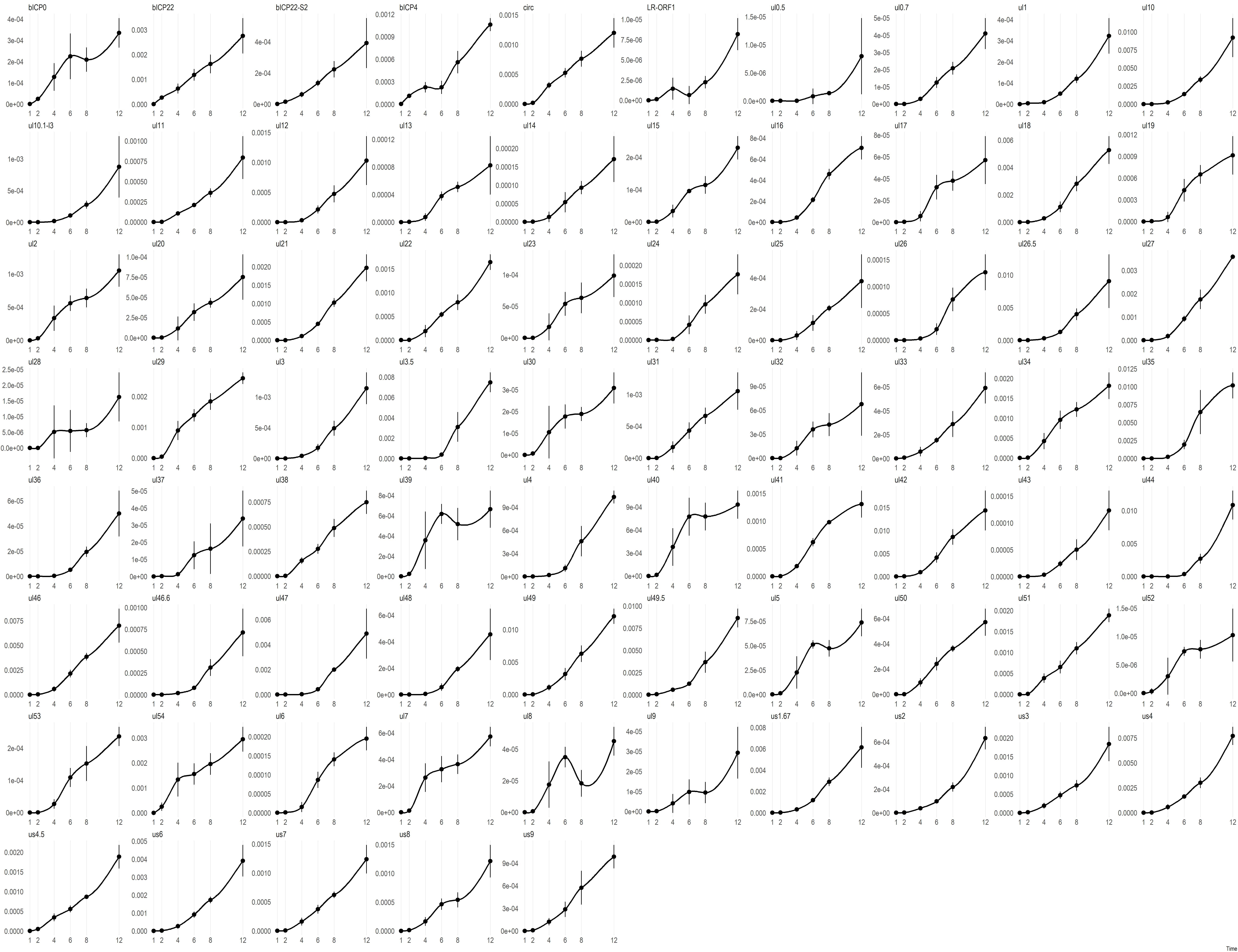

Supplement: Supplementary file 1 [file viruses-14-01289-s001.zip › SupplementaryFigureS5.jpg]

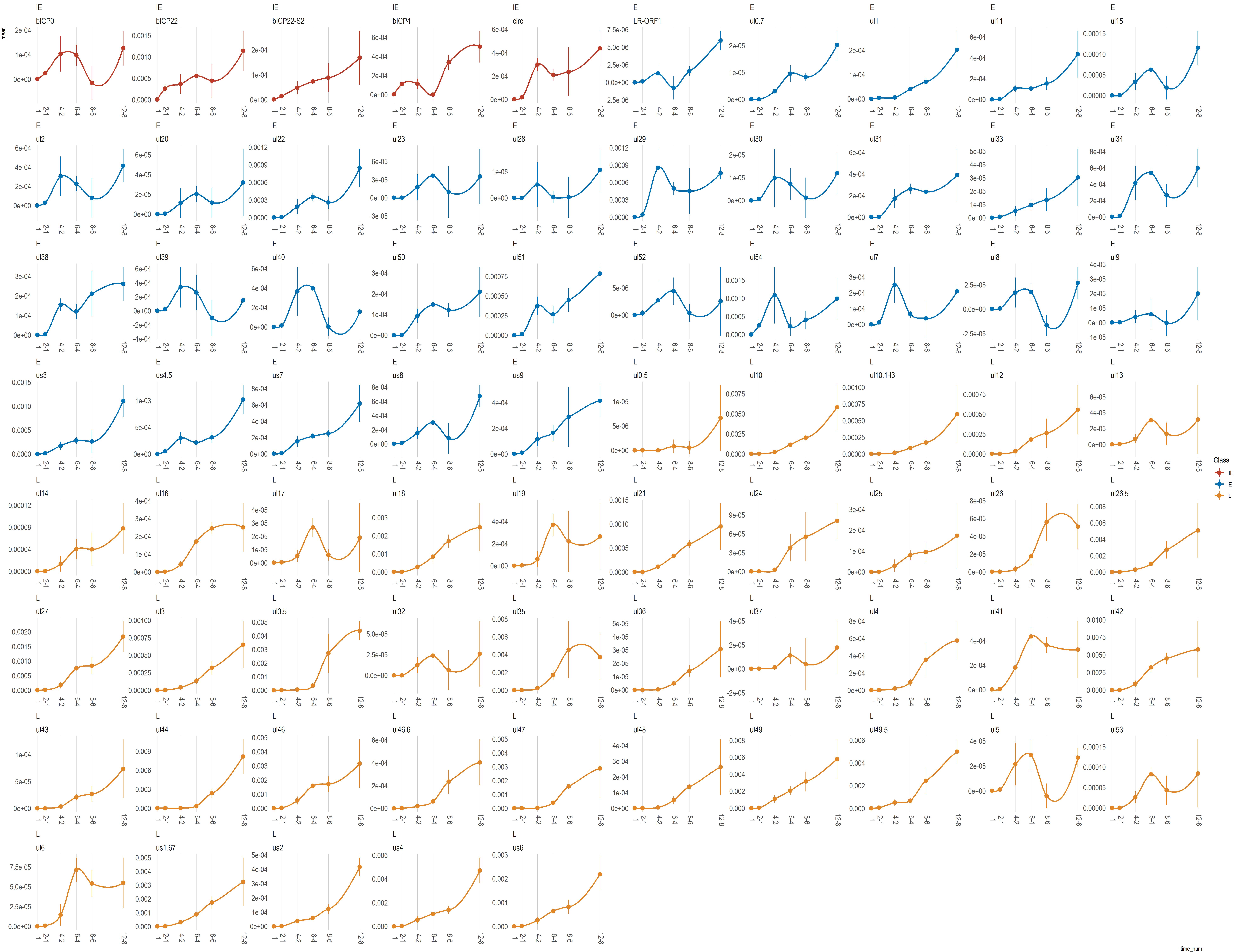

Supplement: Supplementary file 1 [file viruses-14-01289-s001.zip › SupplementaryFigureS6.jpg]

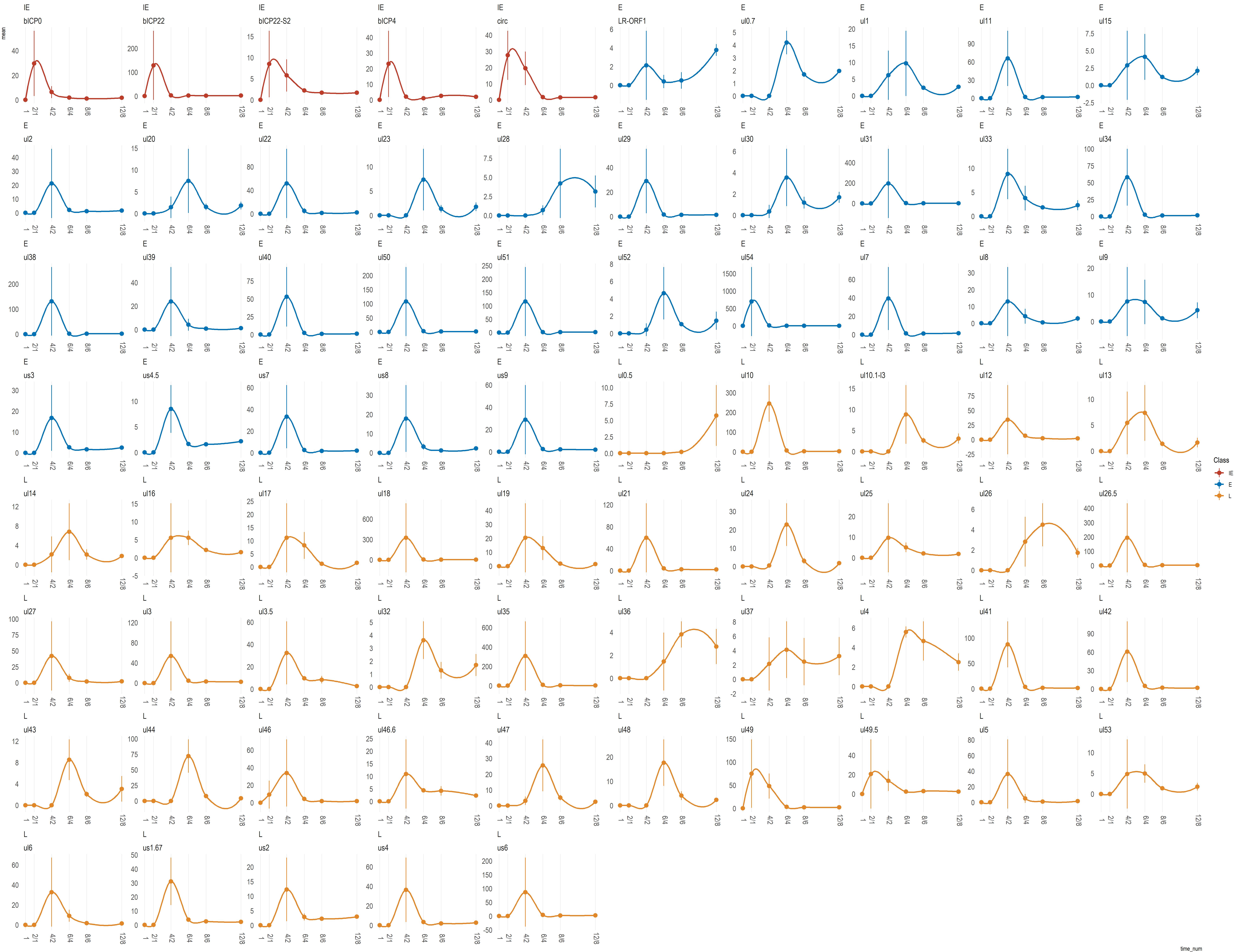

Supplement: Supplementary file 1 [file viruses-14-01289-s001.zip › SupplementaryFigureS7.jpg]

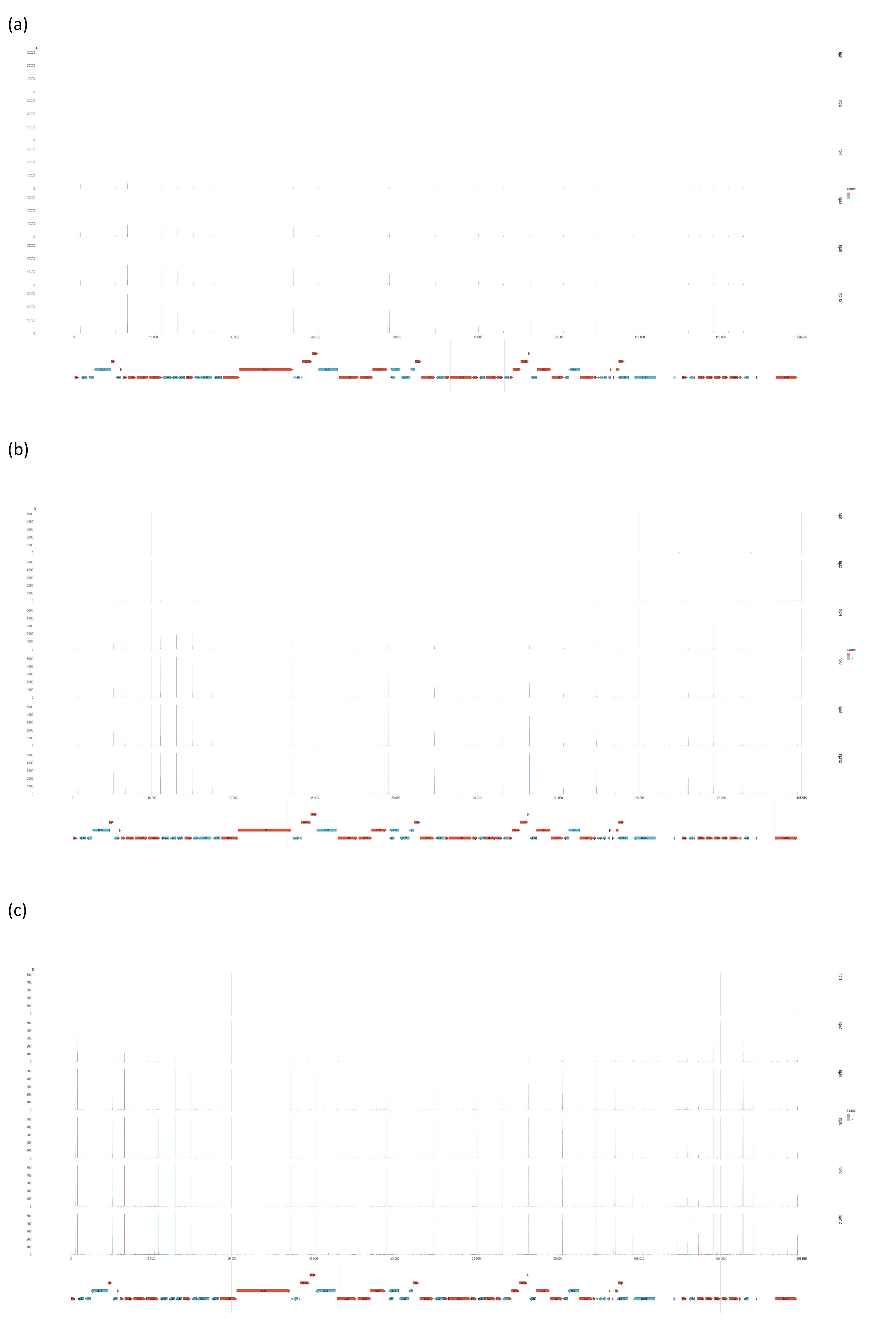

Supplement: Supplementary file 1 [file viruses-14-01289-s001.zip › SupplementaryFigureS8.jpg]
